# Variation in the autism candidate gene *GABRB3* modulates tactile sensitivity in typically developing children

**DOI:** 10.1186/2040-2392-3-6

**Published:** 2012-07-06

**Authors:** Teresa Tavassoli, Bonnie Auyeung, Laura C Murphy, Simon Baron-Cohen, Bhismadev Chakrabarti

**Affiliations:** 1 Department of Psychiatry, Autism Research Centre, University of Cambridge, Cambridge, UK; 2 Current address: Seaver Autism Center, Mount Sinai, NY, USA; 3 Centre for Integrative Neuroscience and Neurodynamics, School of Psychology and Clinical Language Sciences, University of Reading, Reading, UK

**Keywords:** Autism spectrum conditions, autistic traits, GABA, sensory profile, tactile sensitivity

## Abstract

**Background:**

Autism spectrum conditions have a strong genetic component. Atypical sensory sensitivities are one of the core but neglected features of autism spectrum conditions. *GABRB3* is a well-characterised candidate gene for autism spectrum conditions. In mice, heterozygous *Gabrb3* deletion is associated with increased tactile sensitivity. However, no study has examined if tactile sensitivity is associated with *GABRB3* genetic variation in humans. To test this, we conducted two pilot genetic association studies in the general population, analysing two phenotypic measures of tactile sensitivity (a parent-report and a behavioural measure) for association with 43 SNPs in *GABRB3*.

**Findings:**

Across both tactile sensitivity measures, three SNPs (rs11636966, rs8023959 and rs2162241) were nominally associated with both phenotypes, providing a measure of internal validation. Parent-report scores were nominally associated with six SNPs (*P* <0.05). Behaviourally measured tactile sensitivity was nominally associated with 10 SNPs (three after Bonferroni correction).

**Conclusions:**

This is the first human study to show an association between *GABRB3* variation and tactile sensitivity. This provides support for the evidence from animal models implicating the role of *GABRB3* variation in the atypical sensory sensitivity in autism spectrum conditions. Future research is underway to directly test this association in cases of autism spectrum conditions.

## Findings

### Background

Twin studies of autism spectrum conditions (ASC) and autistic traits show high heritability, suggesting a strong genetic component [1,2]. In addition to social and communication deficits, individuals with ASC often show atypical tactile sensitivity, for example, not wanting to wear certain items of clothing [3-5]. However, little is known about mechanisms underlying normative variability in tactile sensitivity. Specifically, the relationship between genotypes and sensory phenotypes is largely unknown. A large scale heritability study on approximately 1,400 twins suggested a moderate genetic influence on tactile sensitivity using parent reports, showing a heritability estimate of 52% [6].

Multiple lines of evidence suggest that the normative variability in tactile sensitivity is related to differences in gamma-aminobutyric acid (GABA) function. For example, heterozygous *Gabrb3* deletion causes increased tactile sensitivity (that is, hypersensitivity) in male mice [7]. In humans, a Magnetic Resonance Spectroscopy study reported that tactile sensitivity, or narrower tactile discrimination thresholds, are significantly associated with GABA levels in the sensorimotor cortex [8]. GABA is a neurotransmitter that is crucial in early cortical development as it has an important role in both excitatory and inhibitory synapses [9,10]. Later in development, GABA is the main inhibitory transmitter in the brain. *GABRB3* codes for one of the principal GABA receptors in the human brain, and is one of the most validated candidate genes for ASC in both humans as well as in animal models [11-13]. Genetic variations within the GABAergic system have been repeatedly associated with ASC and autism-related traits [11-16], though with some non-replications [17]. In view of the strong evidence suggesting an association of *GABRB3* variation with ASC, and specifically a role of *Gabrb3* in tactile sensitivity in mice, we aimed to test variation in this gene for its role in tactile sensitivity in humans.

The current studies report a preliminary investigation of underlying genetic mechanisms of tactile sensitivity in typically developing children. The aim was to test an association between variation in *GABRB3* and two tactile sensitivity phenotypes (measured through a parent-report and a behavioural touch test). The experiment was designed to ensure that the behavioural touch test was identical to that used in mice by DeLorey *et al*. [7] (using the Semmes Weinstein Von Frey Aesthesiometer for touch assessment test). We hypothesized an association between variations in *GABRB3* and tactile sensitivity measured using the touch test. We also hypothesized an association between *GABRB3* variation and tactile sensitivity measured using a tactile subscale of a parent-report, the Short Sensory Profile (SSP). The SSP has been used in ASC, demonstrating differences in tactile sensitivity [18].

To our knowledge, no previous study has investigated if commonly occurring genetic variations have an impact on normative variation in human tactile sensitivity. Understanding sensory perceptual variation in the general population could guide the study of genetic mechanisms underlying sensory issues in ASC.

### Methods

This study was given ethical approval by the National Health Service Suffolk Research Ethics Committee (Reference: 05/Q0102/16).

#### Participants

Signed written consent forms were obtained from 87 parents who then filled in the SSP for their child. Scores were calculated for the SSP tactile subscale (44 boys, 43 girls; children’s mean age: 9.00 years ± 3.22 years). A subset of 39 children took part in the behavioural measured tactile sensitivity study (21 boys, 18 girls; mean age: 11.51 years ± 0.87 years). The Wechsler Abbreviated Scale of Intelligence was used to measure the intelligence quotient (IQ) (mean Full Scale IQ: 117.12 ± 14.38).

#### Short sensory profile (parent-report measure)

The SSP is a caregiver questionnaire with 38 items investigating daily life sensory experiences in children (http://www.pearsonassessments.com). For the current study, the SSP tactile subscale score including seven items was calculated [19]. Internal reliability of the tactile sensitivity subscale ranges from 0.70 to 0.84 [19]. Parents were asked to describe the frequency (1 = always; 5 = never) with which their child performed behaviours such as ‘avoids going barefoot, especially in sand or grass (item 3)’.

#### Touch test (behavioural measure)

Tactile sensitivity was measured using the Semmes Weinstein Von Frey Aesthesiometer for Touch Assessment test (Stoelting Co., Wood Dale, IL, USA), with a forced-choice method of limits paradigm (left or right). Each filament is individually calibrated to deliver its target force within a 5% standard deviation. The participant’s arms were at rest. The experimenter lowered the filaments manually until skin contact was made for 1.5 seconds. The participant was asked to close their eyes throughout. After this, the participant reported where they detected the stimulus by saying ‘left’ or ‘right’. The order was randomized. A ‘method of limits’ procedure with three descending and three ascending trials was applied. The first descending trial started with a thick filament that all participants were able to detect (4.31 g force). Then the next thinnest filament was applied until the participant could no longer detect the stimulus for two consecutive trials. The ascending trial started by increasing the force until the participant could detect the filament in two consecutive trials.

#### Single nucleotide polymorphism selection

Multiple SNPs were selected using the following criteria: minor allele frequency >0.1 in a Caucasian population, and ensuring the maximum gene coverage through linkage disequilibrium [20]. A total of 43 SNPs were chosen, to provide maximal coverage of the gene. DNA was extracted from mouth swabs, which were then anonymized and genotyped using standard PCR-based assays (TaqMan SNP genotyping assays; Applied Biosystems Inc., Foster City, CA, USA). The genotyping call rate was 99.45%. No SNP deviated significantly from Hardy-Weinberg equilibrium (*P* <0.05) (as determined using http://www.changbioscience.com/genetics/hardy).

#### Statistical analysis

UNPHASED 3.1.4 was used to test for genetic association. The following phenotypes were tested for association with all genotyped SNPs (see Additional file 1: Table S1 for all SNPs): parent-reported tactile sensitivity on the SSP, and behaviourally measured tactile sensitivity using a touch test. Bonferroni corrections were used to control for multiple comparisons (i.e. significance level was set to 0.05 and divided by the number of effective comparisons). The effective number of independent genotyped SNPs in *GABRB3* (after accounting for linkage disequilibrium in this sample) is approximately 30, according to the Li and Ji correction [21]. The Bonferroni corrected *P*-value was thus set to 0.001 (<0.05/30).

### Results

#### Descriptive statistics

The parent-report SSP tactile score (32.36 ± 2.80) was in the average range (typical performance for SSP tactile: 35 to 30). High scores on the SSP represent typical performance [19]. Behavioural tactile sensitivity was also in the normal range (mean: 0.06 g ± 0.12 g) [22]. The higher the tactile sensitivity the less sensitive the participant is to touch.

#### Association between *GABRB3* and tactile sensitivity

**Figure 1:**
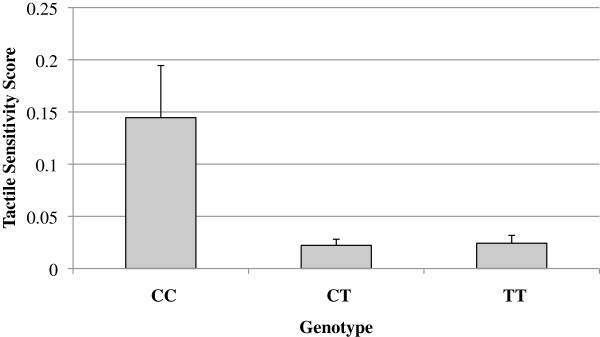
**Association between behaviourally measured tactile sensitivity and rs11636966, a single nucleotide polymorphism in the *GABRB3* gene**. Tactile sensitivity thresholds (g) varied as a function of genotype in this polymorphism. Error bars depict 1 standard error of mean.

Genotypic association (2 degrees of freedom) analysis was conducted using UNPHASED. Both tactile sensitivity measures, that is, the parent-reported SSP tactile score and the behavioural touch test, showed a nominally significant association with three SNPs (rs11636966, rs8023959 and rs2162241) (see Table 1 and Figure 1).

**Table 1:** List of all significant SNPs and their genotypic associations with tactile sensitivity as measured using the parent-report Short Sensory Profile and a behavioural touch test

**Gene variations**	**SSP tactile score (raw score)**				**Behavioural touch test (g)**			
	**mean**	**sd**	**χ² statistic**	** *P* **	**mean**	**sd**	**χ² statistic**	** *P* **
**rs11636966**			10.77	0.004*			10.18	0.006*
C/C	33.34	2.10			0.145	0.187		
C/T	31.25	3.47			0.022	0.024		
T/T	33.00	2.12			0.024	0.021		
**rs8023959**			4.32	0.03*			4.70	0.02*
A/A	32.08	3.06			0.056	0.125		
A/C	33.56	1.75			0.117	0.133		
**rs2162241**			5.68	0.05*			7.07	0.02*
C/C	31.53	3.60			0.021	0.019		
C/T	32.95	2.20			0.087	0.123		
T/T	33.12	1.64			0.141	0.258		
**rs7179514**			10.82	0.004*			4.69	0.09
G/G	32.44	2.97			0.023	0.022		
G/C	31.72	3.06			0.057	0.138		
C/C	34.00	1.78			0.115	0.143		
**rs17117279**			7.75	0.02*			1.72	0.42
A/A	32.69	2.80			0.009	0.136		
A/T	31.96	2.64			0.060	0.115		
T/T	23.00	0.00			0.080	0.000		
**rs7171512**			5.72	0.05*			0.91	0.63
G/G	32.89	2.26			0.096	0.164		
G/A	31.56	3.44			0.050	0.096		
A/A	33.20	2.31			0.027	0.028		
**rs737098**			2.73	0.25			17.75	0.0001**
A/A	31.96	3.46			0.015	0.012		
A/G	32.92	2.03			0.129	0.172		
G/G	32.60	1.52			0.033	0.033		
**rs1367959**			4.43	0.10			13.13	0.001*
C/C	32.62	3.03			0.089	0.143		
C/T	31.65	2.79			0.010	0.005		
T/T	33.55	2.38			0.019	0.015		
**rs1426224**			0.13	0.71			9.78	0.001*
T/T	32.30	3.00			0.040	0.074		
T/C	32.67	2.06			0.215	0.235		
**rs3212331**			3.40	0.18			11.68	0.002*
A/A	32.04	3.19			0.078	0.108		
A/G	32.56	2.60			0.055	0.164		
G/G	33.86	1.86			0.008	0.000		
**rs8026392**			1.42	0.48			11.71	0.002*
T/T	32.05	3.26			0.019	0.019		
T/C	32.62	2.76			0.120	0.171		
C/C	33.13	1.46			0.029	0.026		
**rs11161329**			3.02	0.21			7.29	0.02*
A/A	32.49	2.93			0.053	0.122		
A/T	31.89	3.00			0.032	0.039		
T/T	33.29	2.76			0.138	0.189		
**rs12905535**			3.88	0.14			7.42	0.02*
T/T	31.74	3.55			0.019	0.019		
T/C	32.95	2.00			0.113	0.169		
C/C	32.60	1.52			0.033	0.033		

*P ≤0.05. **P ≤0.001. sd: standard deviation.

Ten SNPs were nominally associated at *P* <0.05 with behaviourally measured tactile sensitivity (see Table 1). After Bonferroni correction for the effective total number of SNPs [21], three SNPs were found to be significant: rs737098, rs1367959 and rs1426224 were associated with tactile sensitivity at *P* <0.001. Six SNPs were nominally associated (at *P* <0.05) with the parent-report SSP tactile sensitivity, none of which survived Bonferroni correction.

### Discussion

This is the first study to test for genetic variation underlying the normative variation in tactile sensitivity in humans. We found that three SNPs in *GABRB3* (rs11636966, rs8023959 and rs2162241) were nominally associated with two measures of tactile sensitivity (a parent-report - the SSP - and a behavioural touch test) suggesting a degree of internal replication. We also found a nominal association between six *GABRB3* SNPs and parent-reported tactile sensitivity on the SSP. Lastly, nominally significant associations between 10 SNPs in the *GABRB3* gene with behaviourally measured tactile sensitivity thresholds were found. After correcting for multiple comparisons, three of these associations remained significant (rs737098, rs1367959 and rs1426224).

These results show that, depending on the specific genotype in certain polymorphisms in the *GABRB3* gene, an individual can have higher or lower tactile sensitivity, measured both through parent-report or a behavioural touch test. This finding is especially interesting because we expected an association based on the study by DeLorey *et al*. [7], which reported that heterozygous knock-out mice for *Gabrb3* have higher tactile sensitivity, that is, they are more sensitive towards touch. In the current study, we used the same behavioural touch test to measure tactile sensitivity and found an association at *P* <0.001 with three SNPs: rs737098, rs1367959 and rs1426224. All of these are intronic in nature.

The observed effects therefore might be mediated through the following mechanisms operating individually or in combination: one or more of the intronic SNPs are in linkage disequilibrium with exonic variation that has a direct effect on gene transcription, and the intronic SNPs themselves might subtly alter the binding affinities for the transcription machinery, thus having an indirect impact on mRNA abundance, structure and/or stability. The exact functional role of these SNPs is however unknown, and can be determined only by directly studying the impact of the different alleles on the expression of the *GABRB3* gene. If the relationship between *GABRB3* levels and tactile sensitivity is equivalent in mice and humans, we can expect that the genotypes associated with high tactile sensitivity would also be associated with reduced *GABRB3* expression. This needs to be tested directly through cell culture studies.

One limitation of the current study is the small sample size, especially for the behavioural touch test. However, we found three SNPs were nominally associated with both tactile sensitivity measures. Furthermore, even though the sample size was particularly small for the behavioural touch test (n = 39), we identified three significant associations after Bonferroni correction. The current studies are nonetheless preliminary in nature, and further data is needed using larger sample sizes.

Another limitation is the lack of an independent replication sample, which is partly due to tactile sensitivity not being a widely used phenotypic measure. Investigations in our laboratory are underway to replicate our findings in a larger sample size. Another limitation of the current work is that we only included typically developing children. It will be important to investigate implications for individuals with ASC in future studies. Our ongoing investigations also include children with ASC.

Little is known about the role of GABA-related effects on sensory perception, and despite its limitations the current study is an important first step. Only one mouse model has investigated the association between *Gabrb3* and tactile sensitivity [7]. These are the first studies looking at an association in humans with relevant phenotypes, which will hopefully stimulate further research in the field. To fully understand underlying causes of atypical sensory sensitivity in ASC, multiple approaches are necessary [1]. Additionally, testing the interplay between environmental and biological influences on sensory sensitivity will be important for future work. The current investigation of the underlying genetic architecture of individual differences in tactile sensitivity opens up the possibility of disentangling the relative contribution of specific genes in different aspects of autistic symptoms.

## Abbreviations

ASC, autism spectrum conditions; GABA, gamma-aminobutyric acid; PCR, polymerase chain reaction; SNP, single nucleotide polymorphism; SSP, Short Sensory Profile.

## Competing interests

The authors declare that they have no competing interests.

## Authors’ contributions

TT, BA, SBC and BC designed the study. BA and TT collected the data. LM, TT and BC carried out the data analyses. All authors were involved in writing the manuscript and approved the final version.

## Supplementary Material

Additional file 1 **Table S1.** List of all SNPs genotyped and their genotypic associations with tactile sensitivity as measured using the parent-report Short Sensory Profile and a behavioural touch test. * denotes significant level of *P* ≤0.05. (PDF 59 kb) Click here for file
