# Supplementary material for: Variation in the autism candidate gene GABRB3 modulates tactile sensitivity in typically developing children
Source: Mol Autism. 2012 Jul 6;3:6. doi: 10.1186/2040-2392-3-6 (PMC3434022; doi:10.1186/2040-2392-3-6)
Supplement: Additional file 1 — Table S1. List of all SNPs genotyped and their genotypic associations with tactile sensitivity as measured using the parent-report Short Sensory Profile and a behavioural touch test. * denotes significant level of P ≤0.05. (PDF 59 kb) [file 2040-2392-3-6-S1.pdf]

Additional Material files

Additional File 1: List of all SNPs genotyped and their genotypic associations with tactile sensitivity as measured using the parent-report Short Sensory Profile (SSP) and a behavioral touch test. * denotes significant level of p≤ .05

Gene variations	SSP tactile score χ^2 statistic	SSP tactile score p-value	Touch test χ^2 statistic	Touch test p-value
rs11636966	10.77	.004*	10.18	.006*
rs8023959	4.32	.03*	4.70	.02*
rs2162241	5.68	.05*	7.07	.02*
rs7179514	10.82	.004*	4.69	.09
rs17117279	7.75	.02*	1.72	.42
rs7171512	5.72	.05*	.91	.63
rs737098	2.73	.25	17.75	.0001**
rs1367959	4.43	.10	13.13	.001*
rs1426224	.13	.71	9.78	.001*
rs3212331	3.40	.18	11.68	.002*
rs8026392	1.42	.48	11.71	.002*
rs11161329	3.02	.21	7.29	.02*
rs12905535	3.88	.14	7.42	.02*
rs6576602	1.34	.51	.01	.92
rs2114485	1.62	.44	4.69	.09
rs1426217	3.46	.17	.36	.83
rs1432007	1.56	.45	1.84	.39
rs10519563	1.47	.22	1.11	.57
rs1248141	5.23	.07	.13	.71
rs2873027	.92	.63	.05	.97
rs9806546	.21	.90	.15	.92
rs12593579	1.36	.50	1.48	.22
rs80838471	2.78	.24	4.76	.09
r10873636	3.43	.17	2.95	.22
rs11631940	1.09	.57	.56	.75

rs12593482	2.88	.14	.66	.71
rs1549482	1.18	.55	4.69	.09
rs17646555	2.38	.30	.56	.75
rs7165604	.22	.63	.71	.45
rs7180158	1.12	.56	.16	.68
rs890317	3.52	.17	4.17	.12
rs12437672	.91	.63	1.25	.53
rs12440905	.35	.55	.49	.48
rs12442889	2.94	.22	2.50	.28
rs21315904	.10	.75	.09	.75
rs7178713	3.65	.16	4.58	.10
rs1863456	2.83	.24	3.90	.13
rs4906896	.92	.62	.01	.90
rs7174437	.02	.86	.24	.62
rs1582760	2.43	.29	1.76	.44
rs1863455	.72	.69	3.18	.20
rs1035751	.96	.32	.14	.70
